# The effects of positive leadership on quality of work and life of family doctors: The moderated role of culture

**DOI:** 10.3389/fpsyg.2023.1139341

**Published:** 2023-03-20

**Authors:** Wei Sun, Xianhong Huang, Xingyu Chen, Yan Wu, Zhen Huang, Yichen Pang, Can Peng, Yunjie Zhang, Hao Zhang

**Affiliations:** ^1^Department of Health Policy and Management, School of Public Health, Hangzhou Normal University, Hangzhou, China; ^2^School of Health Policy and Management, Chinese Academy of Medical Sciences and Peking Union Medical College, Beijing, China

**Keywords:** family doctor, positive leadership, QWL, culture, achievement motivation

## Abstract

**Background:**

Quality of work and life (QWL) of family doctors is highly valued in improving access and equity of healthcare; however, the current low level of QWL in many countries and regions needs to be improved urgently.

**Methods:**

This study explored the effect of positive leadership on the QWL of family doctors, as well as the moderating role of culture, via analysis of data from 473 valid questionnaires of family doctors in China as a sample using SEM, hierarchical linear regression, and a simple slope test.

**Results:**

The empirical results show that positive leadership promoted the QWL of family doctors by improving their achievement motivation and coordinating supportive resources. In addition, our hierarchical linear regression analysis found that the interactive items of positive leadership and culture had a positive effect on achievement motivation (*β*_(a)_  = 0.192), QWL (*β*_(b)_  = 0.215) and supportive resources (*β*_(c)_  = 0.195). Meanwhile, culture had a moderated mediating effect on the relationship between positive leadership and QWL via the achievement motivation of family doctors and supportive resources.

**Conclusion:**

These findings suggest that the interaction among multiple factors, including environmental factors, individual physiological features and culture, may influence the impact of positive leadership on the QWL of family doctors. The possible reasons of these findings and theoretical and practical implications are discussed in this study.

## 1. Introduction

Family doctors, as healthcare gatekeepers provide comprehensive, continuous, and coordinated health services. They have become the key to promoting access and equity in health care. Evidence has shown that family doctor services have achieved significant progress in solving various health problems such as multiple chronic diseases and emerging epidemics, and are especially effective in cost-saving and life-quality improvement under limited healthcare resources (Ferrer et al., 2005). Quality of work and life (QWL) is a key in improving the service quality of family doctors. QWL, first introduced in the 1930s (Che Rose et al., 2006), is an umbrella concept that includes a number of job-related issues that medical staff consider or assess on the job, which in turn have a further impact on the outcome of work (Nowrouzi et al., 2016). Evidence has demonstrated that high levels of QWL, such as reasonable compensation and satisfactory benefits, career expectations, improved interaction among individuals, and high self-efficacy at work, may lead to active work engagement and better service quality (Mohammadi and Ameri Shahrabi, 2013; Srivastava et al., 2019).

Currently, the QWL of family doctors is quite unsatisfactory in many healthcare systems, such as China, one of the largest healthcare systems in the world with inadequate health resources. Meanwhile, in many areas, the lack of resources affects family doctors’ work motivation and leads to their low QWL, which further influences the quality of family doctors’ services. First, the rewards for family doctors, such as compensation and promotion opportunities, are relatively limited. For instance, the 2015 Medscape Physician Compensation Report found that the income gap between family doctors and other specialists remains huge in the United States, and family doctors earn an average of $195,000 annually in comparison with $284,000 for doctors in other specialties (Laff, 2015). In addition, the career expectations of family doctors are not well met. For example, in 2012, 63.5% of family doctors in Changning District of Shanghai City were dissatisfied with their current work (Yuan et al., 2014). In addition, it is very common for family doctors to face an intense working environment, such as long working hours, heavy workloads, and pressure from doctor–patient relationships (Nejatzadegan et al., 2016). The results from a German population-based cohort study reported that the time of family doctor service *per capita* was extremely short due to the heavy workload (Pantenburg et al., 2018). Consequently, the heavy workload may negatively impact the efficiency of doctor–patient communication (Dinkel et al., 2016). Moreover, family doctors have quite low self-efficacy of their job. In an evaluation of service competence in Guangxi Province in China, the general practitioner, the main group of family doctors, only gained an average score of 2.46 out of 5 in a test of services knowledge and skills (Su et al., 2015).

Recently, scholars worldwide have paid close attention to the factors influencing QWL, and there has been a drastic increase in research on the application of positive leadership to improve QWL (Hargett et al., 2017). Positive leadership is a series of leadership strategies that draw on scientific evidence in terms of positive psychology to help leaders develop the medial workforce’s capacity to thrive (Pio, 2021). Positive leadership emphasizes strengths rather than simply focusing on weaknesses; fosters virtuous actions such as compassion, gratitude, and forgiveness; encourages contribution goals in addition to achievement goals; and enables meaningfulness in work (Graves et al., 2013). Increasingly, theoretical and empirical studies have revealed that positive leadership may promote medical staff’s QWL, such as satisfaction of expectations, workplace well-being and self-efficacy, which may further promote the productivity and profitability of work (Gillet et al., 2018). For instance, a study in German hospital surgical departments proved that qualified and efficient leadership was needed to improve doctors’ QWL (Schmitz-Rixen and Grundmann, 2019). A review based on 31 high-quality studies on QWL reported that positive leadership and mindfulness practices will reduce physician burnout, enhance emotional stability and improve cognitive function (Malik and Annabi, 2022).

There might be an important opportunity for developing positive leadership of family doctor service, that is, the development of people-centered integrated care (PCIC). PCIC is a healthcare strategy proposed by the World Health Organization, the two core approaches of which are integration and people-centeredness (The World Bank et al., 2016). PCIC has been promoted in multiple countries and regions, thus providing an environment for developing positive leadership. First, the key approach of PCIC, integration at multiple levels such as organizational integration, resource sharing, and interprofessional collaboration, may provide a bedrock for positive leadership to promote trust and encourage participation (Yuan et al., 2021). For instance, in China, a series of actions guided by positive leadership has been explored, including establishing cross-professional relationships among doctors, nurses, and medical technicians to promote trust, communication and understanding; promoting organizational integration, resource sharing, and cross-professional collaboration such as remote consultation and multidisciplinary team service to leverage the strengths of each participant; and designing a reasonable value-oriented incentive system to encourage contribution. Meanwhile, people-centeredness has also gradually led to a trusting leadership of family doctor teamwork, with a focus on responsiveness, that consciously adopts individuals as participants in, and beneficiaries of trusted team work (Liddle and Ulrich, 2021). People-centered culture has been gradually cultivated through organizational cultural construction, including strategies, goals, guidelines, etc., cultural activities such as publicity and recommendation, and tangible cultural materials such as slogans and banners.

However, it is not clear how positive leadership promotes the QWL of family doctors in the context of PCIC construction. Although some studies have mentioned that the effect of positive leadership on QWL is influenced by multiple factors, such as environmental factors (funding, resources, etc.), individual physiological factors (achievement motivation, etc.), and culture (explicit culture and implicit culture), the complex interactive effect has not yet been clarified. First, most of the existing studies have adopted qualitative research methods rather than sufficient quantitative evidence. In addition, influencing factors are often simply screened, while the interactions among multiple factors have not yet been clarified. Above all, very few studies have analyzed the impact of positive leadership in the context of PCIC, although PCIC is an important global health strategy. Therefore, this study aims to explore the influence of positive leadership on the QWL of family doctors in China by clarifying the interaction among multiple influencing factors, including environmental factors, physiological factors, and culture in the context of PCIC.

## 2. Theoretical basis

### 2.1. Positive leadership and QWL of family doctors

QWL is an umbrella concept that includes a number of job-related issues including work reward, work environment, work-life balance and self-efficacy. Recent studies suggest that positive leadership may have a direct positive effect on QWL. For instance, during the epidemic in America, sufficient positive leadership in medical service improved healthcare providers’ perceived professional identity and work reward (Ness et al., 2021). A study from the Mayo Clinic reported that support of positive leadership was conducive to improving the work environment and motivating higher self-efficacy of US physicians to some extent (Shanafelt and Noseworthy, 2017). A study in Switzerland also proved that high-quality leadership reduced the work stress of doctors in acute and rehabilitation hospitals by improving their work environments and promoting their work-life balance (Peter et al., 2021).

Given that positive leadership may have a positive effect on QWL, the following hypothesis is proposed for this study:

*H1*: Positive leadership has positive effects on QWL.

### 2.2. The mediating roles of individual physiological factors between positive leadership and QWL

Previous studies have shown that positive leadership may be associated with achievement motivation, one of the most important factors in psychology. Achievement motivations are defined as the internal drivers of individuals to pursue valuable goals, achieve high performance, and strive for success (Elliot et al., 2016). As proof, a study in Harbin City in China proposed that leadership improved the psychological quality of doctors and promoted them to pursue more extrusive achievements, contributions, honor and social status (Chu et al., 2021). Research from 17 government hospitals in Pakistan showed that positive leadership improved nurses’ job motivation to achieve high performance (Asif et al., 2019). A study from Morocco proved that positive leadership enhanced health workers’ willingness to pursue public service values, which further promoted their achievement motivation (Belrhiti et al., 2020).

In addition, achievement motivation is possibly related to QWL, such as work expectation, self-efficacy, and work-life balance. For instance, a study from 11 community healthcare centers (CHCs) in Zhenjiang Province, China proved that general physicians with higher achievement motivation achieved better self-efficacy and work expectations (Ge et al., 2022). A study about burnout syndrome in Spain, Ceuta, demonstrated that health workers with greater achievement motivation scored higher, which may be related to their higher demand for efficacy and work expectations (Domínguez Fernández et al., 2012). A study conducted in the southeastern region of Nigeria confirmed that tuberculosis management medical workers’ motivation to finish work tasks was related to their high sense of work-life balance (Ogbuabor and Okoronkwo, 2022).

By integrating these findings, we may conclude that positive leadership could positively influence QWL through achievement motivation. Thus, the following hypotheses are proposed for this study:

*H2*: Positive leadership has positive effects on achievement motivation.

*H3*: Achievement motivation has positive effects on QWL.

### 2.3. The mediating roles of supportive resources between positive leadership and QWL

For healthcare decision makers, supportive resources are a pivotal challenge (Smith et al., 2013). Prior research has verified that supportive resources may be associated with positive leadership, and evidence has also proven that positive leadership development is essential to address resource inequalities in health care (Pascal et al., 2017). For example, a summary of the experience from the American Human Resource Management Program noted that positive leadership helped leaders to coordinate various departments and optimize their functions to solve human resource crises in healthcare (Sherk et al., 2009). A literature review of human capital resources suggested that managers with positive leadership were able to coordinate the organizational human resource management process and strengthen the performance assessment system related to financial distribution (Eckardt et al., 2020). Research on emergency management in elderly healthcare from America confirmed that rapid coordination of intensive care beds and reasonable allocation of ventilator use during the epidemic relied on physicians’ emergency response capacity of positive leadership (Aitken and Ibrahim, 2021).

Additionally, previous studies have revealed that physicians’ QWL may be improved by supportive resources, including health human resources, financial input and medical equipment resources. For example, proof from Polish hospitals suggested that human resource management was related to the working efficacy of physicians and doctor–patient relationships (Storman et al., 2021). A study in Nepal attested that material resource support is associated with doctors’ financial satisfaction (Vaidya et al., 2020). Research on physicians’ career retention from Tanzania proved that general physicians’ work environment and career expectations were improved by increasing financial resource investment (Sirili et al., 2018).

Moreover, evidence also proved that reasonable supportive resources may benefit the achievement motivation of doctors. For example, doctors from 30 different cities in China mentioned that supportive resources of clinical practice made them maintain greater comfort and psychological security, thus boosting their positive emotions, stability of work and personal goal (Zhang et al., 2021). A qualitative study from Abbottabad, Pakistan, showed that physicians’ work motivation was related to adequate remuneration, supplies and medical facilities (Shah et al., 2016).

Integrating these findings, positive leadership may have a significant effect on QWL through the chain mediation of supportive resources and achievement motivation. As a result, the following hypotheses are proposed for this study:

*H4*: Positive leadership has positive effects on supportive resources.

*H5*: Supportive resources have positive effects on QWL.

*H6*: Supportive resources have positive effects on achievement motivation.

### 2.4. The moderating role of organizational culture

**Figure 1:**
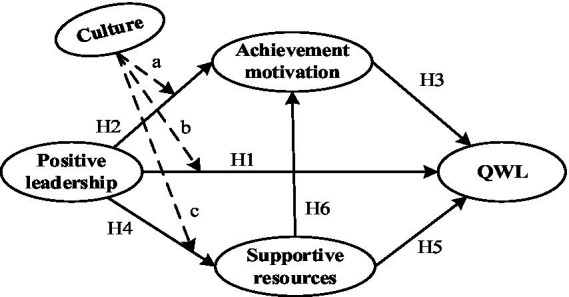
Research model diagram of promoting the QWL of family doctors.

Organizational culture is defined as the values and beliefs of the organizational environment (Schneider et al., 2013). Previous studies have revealed that culture is important to improve the development of organizations and, in turn, to improve workers’ QWL in hospitals. As an example, a study from two private hospitals in the city of Madiun, Indonesia, testified that a positive organizational culture helped medical physicians feel satisfied with the work environment and boosted their active work engagement (Srimulyani and Hermanto, 2022). A national survey in America during the pandemic proved that organizational culture had an effect on work expectations and achievement motivation among physicians (Young et al., 2020). In addition, research at Consultant Company in Indonesia proved that sharping a positive organizational culture changed employees’ attitudes toward work and thus improved their QWL (Fakhri et al., 2020). A study in Korea certified that positive culture support was the key to relieving employees’ perceived fatigue and improving self-efficacy and ultimately had a positive impact on their QWL (Kim and Jang, 2018). Therefore, we predict that culture may facilitate the influence of QWL (Figure 1).

## 3. Materials and methods

### 3.1. Participants and data collection

The data used in this study were collected in China. The Chinese government begun exploring the family doctor services as early as the 1980s and has recently committed to developing a contract-based family doctor service system. In 2016, the State Council Medical Reform Office issued the *Opinions on Promoting Family Doctors’ Contracting Services*, which aimed to establish a contract-based family doctor service system in a CHC, with family doctor teams composed of Western doctors, traditional Chinese medicine doctors, nurses, medical technicians, public health doctors, etc. (China State Council Healthcare Reform Leading Group et al., 2016). Family doctor service is likely to touch on a wide range of people and it is estimated to be provided to at least 75% of China’s population by 2035. Thus, it is urgently needed to improve the QWL of family doctors.

Zhejiang Province was selected as the sampling area for two reasons. First, family doctor services have a wide impact on the residents of Zhejiang Province, since Zhejiang Province is one of the first regions in China to explore family doctor services, and more than 85% of key groups, including elderly and chronic patients, have signed service contracts with family doctors. The government has implemented a series of policies to develop family doctor services, including measures aimed at improving service capacity and quality. Second, the reform of the PCIC has had a great impact on Zhejiang Province. In 2016, the World Bank Group, the World Health Organization, and the China Health Planning Commission jointly issued the *Report on Deepening the Reform of China’s Medical and Health System*, which suggested that China should take steps in practicing PCIC, and Zhejiang Province was one of the earliest regions to carry out PCIC in China. Hangzhou City and Changxing County of Zhejiang Province were selected as sample areas for the WHO to evaluate the development level of PCIC in China. With a *per capita* GDP of 113,000 yuan in 2021, Zhejiang Province has an advanced economic status. By the end of 2021, more than 27 thousand family doctors in Zhejiang had signed service contracts with 24.49 million residents.

Inclusion criteria for voluntary participation in this study were as follows: (1) offered informed consent; (2) worked in CHCs in Zhejiang Province and provided family doctor service for more than six months; and (3) aware of QWL of the family doctor service. Exclusion criteria were as follows: (1) had no contract with residents for family doctor services; (2) provision of family doctor service was no more than six months; (3) trainee medical staff, medical staff from hospitals temporarily providing services in CHCs.

We determined the sample size according to the principle of structural equation model (SEM). Thompson B suggested that the ratio of the number of people to the number of measured variables in SEM should be at least 10:1, and 15:1 or 20:1 are much better (Thompson, 1998). Since there are 21 measured variables in the SEM, 420 respondents is sufficient in theory, and in practice, 500 questionnaires were distributed to family doctors between July 1 and November 30, 2021, in which 473 questionnaires qualified, and sample effective rate 94.6%. Sampling regions were selected with judgment sampling on the basis of economic development. We stratified counties and districts in Zhejiang Province into developed regions, developing regions and underdeveloped regions by systematic clustering analysis and took indicators into consideration, such as regional economic indicators (gross domestic product, total retail sales of consumer products, etc.), public finance indicators (total fiscal revenue, general public budget revenue, etc.), and medical and health services indicators (the number of medical institutions, the number of accommodations, the number of health technicians, etc.) On the basis of the above cluster analysis, we selected Hangzhou as the representative of developed areas, Lishui as the representative of underdeveloped areas, and Jiaxing Nanhu District, Pinghu County, Jiaxing Port District, and Huzhou Changxing County as the representatives of developing areas. With convenience sampling, 20 family doctors were chosen from medical institutions in each district. This study carried out a formal survey after a pilot study was applied and was modified. Then, the exclusion criteria for questionnaires were as follows: (1) omissions in the answer; (2) more than half of the responses were the same; and (3) similar answers to apparently contradictory questions. If the questionnaire met any of these criteria, it was excluded. We provide the variance-covariance matrices of this study (Supplementary material).

### 3.2. Measurement

A questionnaire was exploited for this study using the following steps: first, measurement indicators including positive leadership, supportive resources, achievement motivation and QWL, were screened through a literature review; second, the indicators draw from the literature was translated to Chinese language. The indicator which needed to be translated mainly included the Positive Leadership scale (Meijer et al., 2018), supportive resources scale (Lukes and Stephan, 2017), Achievement Motivation Scale (Smith et al., 2019). However, the initial indicators which were Chinese version in previous studies needed no translation, for instance, the Brooks QWL questionnaire (Brooks and Anderson, 2004) and the General Self-Efficacy Scale (Schwarzer et al., 1999). A series of measurements were taken to insure the accuracy of translation. For example, the indicators was translated by professional translation software, and then checked by native Chinese speakers whose major is English. A preliminary survey was also conducted to encourage participants to express their perceptions of the indicators, and further to ensure that the questionnaire will be able to fully and accurately achieve the measurement objectives; third, telephone interviews were conducted with 70 medical staff members, the visit records were analyzed in NVivo (11th edition) on the basis of grounded theory, and the evaluation indicators were summarized; fourth, two rounds of Delphi consultation were carried out with 22 scholars, medical staff and health administrators, and the measurement indicators were optimized; and fifth, the questionnaire was further modified and verified by a pilot study. Internal consistency (Cronbach’s α coefficient) and composite reliability (CR) were used to evaluate the reliability of the questionnaire, and then the convergent validity and heteroplasm-elemental ratio were used to assess the validity of the questionnaire. The questionnaire comprised the following: general information, positive leadership scale, supportive resources scale, achievement motivation scale, QWL scale, and culture scale.

#### 3.2.1. General information

The form for general information comprised items pertaining to gender, age, marital status, educational level, post, income, work shift, and work seniority.

#### 3.2.2. Positive leadership

The positive leadership scale was conducted to assess whether the leaders of healthcare institutions coordinated and motivated the development of family doctor services. We extracted the initial indicators from the literature, including a questionnaire about leadership designed by Meijer (Meijer et al., 2018) and the findings of different leadership forms (D’Innocenzo et al., 2016). We also distilled indicators through thematic analysis to optimize the indicators, with a total word frequency of 60. The indicators were acquired through two rounds of Delphi consultation, with an average score for importance over 4 and an average score for availability above 3.5. The indicators consist of 3 items: organization of positive leadership, coordination of positive leadership and incentive of positive leadership. All items were rated on a seven-point Likert scale, ranging from 1 = fully disagree to 7 = fully agree. The internal consistency of this scale was satisfactory with a Cronbach’s alpha of 0.977, CR of 0.979, AVE of 0.941, and the heteroplasm-elemental ratio was less than 0.90.

#### 3.2.3. Supportive resources

The supportive supportive resources scale was conducted to estimate whether the human resources, funding and material resources of family doctor services are sufficient. We extracted the initial indicators from the literature, including the Innovation Support Scale (ISI) (Lukes and Stephan, 2017) and the organizational environment framework validated by Meijer et al. (2018). We further optimized the indicators through thematic analysis, with a total word frequency of 129. The indicators were obtained through the two rounds of Delphi consultation, with an average score for importance over 4 and an average score for availability above 3.5. The indicators consist of 3 items, human resources, financial support and material resources, and a seven-point Likert scale was used to measure each item, ranging from 1 = fully disagree to 7 = fully agree for the scale. The scale demonstrated high interitem consistency with a Cronbach’s alpha of 0.977, CR of 0.936, AVE of 0.834 and the heteroplasm-elemental ratio was less than 0.90.

#### 3.2.4. Achievement motivation

The achievement motivation scale was conducted to evaluate family doctors’ intrinsic motivation to provide family doctor services. We extracted the initial indicators from the literature, including the Achievement Motivation Scale (AMS) (Smith et al., 2019) and the revised 10-item version of the AMS (Lang and Fries, 2006). Then, we further enriched the indicators of achievement motivation. The indicators consist of 3 items, including interest, work attraction and concern about work, with a seven-point Likert scale measurement, ranging from 1 = fully disagree to 7 = fully agree. The achievement motivation scale was qualified, with a Cronbach’s alpha of 0.866, CR value of 0.897, and AVE value of 0.750 (Tables 1, 2).

**Table 1:** Measurement items and results of reliability and validity analysis of the questionnaire (*N* = 473).

Construct	Dimension	Measurement items	Load^a^	Cronbach’α	CR	AVE
Positive leadership		A1 Family doctor services are organized by positive leadership.	0.975	0.977	0.979	0.941
		A2 Teamwork are coordinated and interpersonal relationship are promoted by positive leadership.	0.986			
		A3 Team members are motivate to participate actively by positive leadership.	0.948			
Supportive resources		B1 There are sufficient human resources involved in the family doctor services.	0.901	0.936	0.938	0.834
		B2 Family doctor services are well funded by the government.	0.923			
		B3 There are sufficient resources to support family doctor services.	0.916			
Achievement motivation		C1 I am interested in family doctor services.	0.928	0.886	0.897	0.750
		C2 I am attracted by the challenge of the family doctor services.	0.975			
		C3 I care if I am qualified for family doctor services.	0.661			
QWL	Reward	D1 I am satisfied with the compensation of family doctor services.	0.872	0.939	0.940	0.840
		D2 I am given the chance to gain honorary title through family doctor services.	0.939			
		D3 I gain promotion opportunities or learning opportunities through family doctor services.	0.937			
	Expectation of work	E1 I can realize my life value though family doctor services.	0.825	0.912	0.918	0.790
		E2 I am optimistic about the development of the family doctor services.	0.927			
		E3 My expectation for work is satisfied by family doctor services.	0.911			
	Working environment	F1 Family doctor services are supported by preferential medical insurance.	0.870			
		F2 Family doctor services are supported by friendly health laws, regulations and policies.	0.875	0.889	0.891	0.732
		F3 Family doctor services are supported by harmonious doctor–patient interaction environment.	0.821			
	Self-efficacy	G1 I am qualified to serve as a family doctor if I make my effort.	0.900			
		G2 I can find the solution when I encounter difficulties in family doctor services.	0.954	0.913	0.916	0.786
		G3 I have confidence in my skills in family doctor services.	0.799			
Culture		H1 People-Centered organizational strategies, targets, guidelines, etc., for family doctor services.	0.975	0.964	0.964	0.899
		H2 People-Centered cultural activities such as publicity and commendation for family doctor services.	0.942			
		H3 People-Centered tangible materials such as slogans, banners and brochures for family doctor services.	0.927			

^a^All loading values are significant at the 0.001 level. CR, composite reliability; AVE, average variance extraction.

**Table 2:** Heterotrait – Monotrait (HTMT) of the questionnaire (*N* = 473).

HTMT	F1	F2	F3	F4	F5	F6	F7	F8
Positive leadership	—							
Supportive resources	0.783	—						
Achievement motivation	0.768	0.821	—					
Reward	0.796	0.881	0.808	—				
Expectation of work	0.851	0.845	0.970	0.892	—			
Working environment	0.870	0.885	0.878	0.848	0.908	—		
Self-efficacy	0.813	0.891	0.921	0.853	0.930	0.912	—	
Culture	0.893	0.772	0.807	0.822	0.922	0.859	0.829	—

#### 3.2.5. QWL

The QWL scale was conducted to evaluate the family doctor’s perception of work, including working reward, working expectation, working environment and self-efficacy. Of them, working rewards refer to whether family doctors are provided with satisfying compensation and opportunities for career development. Work expectation refers to whether family doctors achieve professional value and personal growth through their work; working environment refers to whether family doctor services are supported by a favorable and friendly environment; and self-efficacy refers to family doctors’ confidence level in their capability. We extracted the initial indicators from the Brooks QWL questionnaire (Brooks and Anderson, 2004) and the General Self-Efficacy Scale (GSES) (Schwarzer et al., 1999). Then, because the items extracted from these scales did not absolutely match our measurement goals and method programs, we adjusted the QWL scale to some extent. The four dimensions of QWL are measured by twelve items, with a seven-point Likert scale measurement, ranging from 1 = fully disagree to 7 = fully agree. Each dimension of the QWL scale was qualified, with a Cronbach’s alpha of 0.939, 0.912, 0.889, and 0.913.

#### 3.2.6. Culture

The culture scale was conducted to assess whether people-centered culture was developed for family doctor services. We extracted the initial indicators from the organizational culture conception in the Innovation Support Scale (Lukes and Stephan, 2017) and further optimized the culture scale. The indicators consist of 3 items, including organizational culture, cultural activities and tangible cultural materials, with a seven-point Likert scale measurement, ranging from 1 = fully disagree to 7 = fully agree. The culture scale was qualified, with a Cronbach’s alpha of 0.964, CR value of 0.964, and AVE value of 0.899.

### 3.3. Quality control

After designing the questionnaire, we ran a preliminary test. We asked the participants who took part in the preliminary test about their perceptions of the questionnaire to improve it. A pilot questionnaire survey with a sample size of 200 was conducted, the reliability and the validity of the questionnaire was evaluated. During the whole process of the pilot study, each participant was informed the purpose and method of the study. In addition, the questionnaires were distributed by graduate students with survey experience. Prior to the formal survey, standardized training was provided to the investigators to ensure that they had a clear understanding of the survey content and that they followed unanimous standards and methods. They administered the investigation by distributing the paper questionnaire to the family doctors who met the inclusion criteria. Each participant completed a questionnaire independently, with investigators available to address the questions. In the questionnaire, participants were asked to fill in their cell phone number and ID number to match the research data obtained from the survey. This information served to identify and match identities under conditions of anonymity. After data collection was completed, the questionnaires were examined by the investigators, and those that did not meet the study requirements were checked with the participants. Subsequently, the questionnaires were coded and the data were double-entered.

### 3.4. Ethical considerations

Following the ethical guidelines outlined in the 1964 Declaration of Helsinki and its later amendments, data collection was carried out with the approval of the Ethics Committee of Hangzhou Normal University (approval number: 20190022). During the survey, each participant gave informed consent and understood the purpose and methods of the study. Due to the principle of privacy protection, this study stated that participants’ information will only be used for research purposes.

### 3.5. Statistical analysis

#### 3.5.1. Preliminary analyses

Statistical packages including Analysis of Moment Structure (Amos) version 24.0, Statistical Product and Service Solutions (SPSS) version 26.0 and the macro PROCESS procedure for SPSS version 4.1 were applied to analyze the data in this research. Normal distribution, outliers, and multicollinearity were evaluated through the following standards: first, normal distribution was examined by kurtosis (ku) and coefficients of skewness (sk); second, the presence of outliers was determined by Mahalanobis Distance; third, multicollinearity was measured through the variance inflation factor (VIF) and tolerance rate; fourth, correlations of main variables were reported by Spearman correlations (shown as values of *r*).

#### 3.5.2. Mediation and moderation analyses

We used SEM to analyze the mediating effect of supportive resources and achievement motivation. To evaluate the reliability of the questionnaire, we employed composite reliability (CR) and internal consistency (Cronbach’s coefficient). To assess the validity of the questionnaire, we employed convergent validity (average variance extracted, AVE) and discriminant validity (heterotrait-monotrait, HTMT). Additionally, we implemented the maximum-likelihood method in the SEM to assess and validate the effecting path from positive leadership, supportive resources and achievement motivation to QWL. In succession, we applied 5,000 replicate samplings of the percentile bootstrap method at the 95% confidence level (CI) to measure the mediating effect, and if the value of CI did not include 0, the difference in effect had statistical significance. In addition, we employed hierarchical linear regression analysis in SPSS to examine the moderating effect of culture on the impact of positive leadership on achievement motivation, positive leadership on QWL and positive leadership on supportive resources. To further test the positive moderating effect, we applied a straightforward slope test. Ultimately, Model 85 in the SPSS PROCESS macro was used in the model with positive moderating effect to test the moderated mediation effect of culture in the path of positive leadership on QWL, with supportive resources and achievement motivation as two mediators. When the culture score was the sample mean and at plus or minus 1 SD, we tested the indirect moderated mediation of positive leadership on QWL through supportive resources and achievement motivation.

## 4. Results

### 4.1. Demographic characteristics

The results of descriptive analysis and QWL scores are shown in Table 3. Among the 473 total participants, 353 (74.6%) were female, and 120 (25.4%) were male. In terms of age, the participants were concentrated in the age group of 25–35 (34.9%), followed by those in the age group of 35–45 (38.1%). In terms of marital status, 293 (61.9%) respondents were married. For education level, 328 (69.3%) respondents had a college degree, and 84 (17.8%) respondents had a junior college degree. Regarding posts, the respondents were mainly Western medicine doctors (49.7%) and nurses (9.1%). In terms of income, the respondents were mainly in the high-income group (62.6%). For working shifts, 256 (54.1%) of the respondents were on regular shifts, and 217 (45.9%) were on shifts. Finally, 120 (25.4%) had worked 20 years or more. The QWL of family doctors was assessed by scale scores. There were significant differences in respondents’ QWL scores across age (*F* = 10.60, *p* < 0.01), marital status (*F* = 51.89, *p* < 0.01), educational background (*F* = 9.20, *p* < 0.01), post (*F* = 10.28, *p* < 0.01), and income (*F* = 96.60, *p* < 0.01). Youthful (<25) family doctors (*M* = 5.89, SD = 0.98) had the highest score. Married (*M* = 5.29, SD = 1.05) family doctors had the highest score. The participants with a high school degree and below (*M* = 5.30, SD = 1.00) scored highest; however those with a master’s degree and above (*M* = 3.17, SD = 1.19) had the lowest score. High-income (≥50,000) respondents (*M* = 5.36, SD = 1.01) had the highest score, and low-income (≤10,000) respondents (*M* = 3.41, SD = 0.82) scored the lowest. Medical technicians (*M* = 5.50, SD = 0.79) had the highest score, while western medicine doctors (*M* = 4.45, SD = 1.34) scored the lowest. Except for those aforementioned, gender (*t* = 0.695, *p* = 0.60), work shift (*t* = 1.07, *p* = 0.65) and work seniority (*F* = 2.81, *p* = 0.25) were not significant in the difference in QWL score.

**Table 3:** Comparison of family doctors’ mean QWL scores on the questionnaire based on different demographic variables (*N* = 473).

Characteristic	Categorization	Total score
Gender	Male (120)	4.87 ± 1.33
	Female (353)	4.77 ± 1.29
	*t* (*p*)	0.695 (0.60)
Age (years)	<25 (35)	5.89 ± 0.98
	25- (165)	4.85 ± 1.26
	35- (180)	4.60 ± 1.34
	45- (93)	4.68 ± 1.20
	*F* (*p*)	10.60 (<0.01)
Marital status	Unmarried (163)	3.98 ± 1.26
	Married (293)	5.29 ± 1.05
	Divorced (9)	3.35 ± 0.77
	Other (8)	4.80 ± 1.30
	*F* (*p*)	51.89 (<0.01)
Educational background	High school and below (38)	5.30 ± 1.00
	Junior college (84)	5.08 ± 1.23
	College (328)	4.74 ± 1.30
	Master and above (23)	3.71 ± 1.19
	*F* (*p*)	9.20 (<0.01)
Post	Western doctor (235)	4.45 ± 1.34
	Chinese traditional medicine doctor (43)	4.57 ± 1.36
	Public health doctor (26)	5.33 ± 0.86
	Nurse (118)	5.16 ± 1.18
	Medical technician (36)	5.50 ± 0.79
	Others (15)	5.49 ± 0.98
	*F* (*p*)	10.28 (<0.01)
Income	low (131)	3.41 ± 0.82
	lower–middle (5)	3.97 ± 1.16
	middle (8)	5.13 ± 1.16
	upper–middle (33)	5.31 ± 1.00
	high (296)	5.36 ± 1.01
	*F* (*p*)	96.60 (<0.01)
Work shift	Regular (256)	4.86 ± 1.31
	Shift (217)	4.73 ± 1.28
	*t* (*p*)	1.07 (0.65)
Work seniority	<5 (53)	5.31 ± 1.41
	5- (111)	4.78 ± 1.26
	10- (104)	4.84 ± 1.26
	15- (75)	4.65 ± 1.31
	20- (130)	4.65 ± 1.26
	*F* (*p*)	2.81 (0.25)

Means, standard deviations, and Spearman correlations among the variables are presented in Table 4, since the data are non-normally distributed. An examination of the correlations indicates that the correlations among all variables (*p* < 0.01) were significant, which includes positive leadership, supportive resources, achievement motivation, QWL and culture.

**Table 4:** Mean, standard deviation, and correlation coefficient of each variable (*N* = 473).

Variable	1	2	3	4	5
1. Positive leadership					
2. Supportive resources	0.755^**^				
3. Achievement motivation	0.746^**^	0.768^**^			
4. QWL	0.858^**^	0.874^**^	0.869^**^		
5. Culture	0.852^**^	0.741^**^	0.755^**^	0.873^**^	
Mean value	5.125	4.451	4.853	4.7985	5.153
Standard deviation	1.4308	1.5050	1.3751	1.2972	1.332

^**^p < 0.01, two-tailed test.

### 4.2. Construction and fit of the SEM

In this study, SEM was established with positive leadership, supportive resources and achievement motivation as independent variables and QWL as the dependent variable. We applied the maximum-likelihood method to evaluate the initial model. The C.R. value in essence represents the normalized estimation of multivariate kurtosis (Mardia, 1970). According to Bentler (2005), values >5.00 shown in practice, are usually a symbol that the data are non-normally distributed. The *z*-statistic was 292.505, which indicates non-normality of the data. Thus, to accommodate the lack of multivariate normality, we applied the Bollen-Stine bootstrap method to adjust the model and parameters (Bollen and Stine, 1992). Table 5 shows the results of the acceptable fit for the measurement model with the modified index.

**Table 5:** Fitting results of the structural equation model.

Fitting index	Fitting standard	Model	
		Initial model	Modified model
Chi-square freedom ratio χ^2^/*df*	1 < χ^2^/*df* < 3 Good	3.620	1.612
Root mean square error of approximation RMSEA (90%CI)	<0.05 Good	0.053	0.036
Goodness of fit index, GFI	>0.90 Good	0.844	0.964
Adjusted goodness of fit index, AGFI	>0.90 Good	0.799	0.950
Normed fit index, NFI	>0.90 Good	0.920	0.964
Confirmatory fit index, CFI	>0.90 Good	0.940	0.986
Bentler and Bonett’s non-normed fit index, TLI	>0.90 Good	0.930	0.984

**Figure 2:**
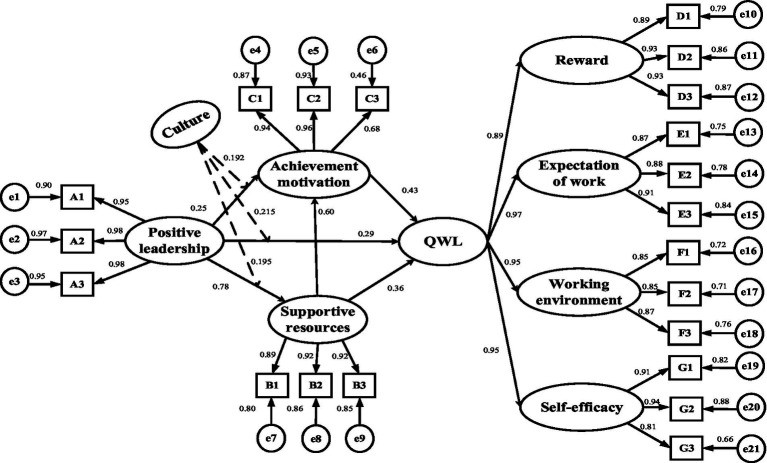
Model diagram of the influence of positive leadership promoting QWL.

Figure 2 is the demonstration of the definitive model, which indicates the standardized path coefficients for the whole model. The results of the causal model proved that: (1) the effects of positive leadership on QWL, achievement motivation and supportive resources were significant, with standardized path coefficients of 0.29, 0.25 and 0.78 (*p* < 0.001), respectively; therefore, H1, H2 and H4 were verified; (2) the effects of supportive resources on QWL and achievement motivation were significant, with standardized path coefficients of 0.29 and 0.60 (*p* < 0.001), respectively; thus, H3 and H6 were supported; and (3) the effect of supportive resources on QWL was significant, with a standardized path coefficient of 0.43 (*p* < 0.001); accordingly, H5 was supported.

### 4.3. Analysis of simple mediating effect

We employed a percentile bootstrap approach to assess whether the three mediating effects demonstrated in the SEM were significant, which is concretely reflected in Table 6. First, the significant indirect effect of positive leadership on QWL *via* the mediation of supportive resources was 49.2% of the total effect (the 95% CI was 0.192–0.350). Because the 95% CI did not include 0, the direct effect of this path was as well as prominent (the 95% CI was 0.200–0.353), showing that supportive resources partially mediated the effect of positive leadership on QWL. In line with this result, the mediation of achievement motivation was significant (the 95% CI was 0.043–0.173) and occupied 27.1% of the total effect, revealing that achievement motivation also incompletely mediated the effect of positive leadership on QWL. Additionally, the significant indirect effect of positive leadership on QWL *via* the chain mediation of supportive resources and achievement motivation accounted for 40.7% of the total effect (the 95% CI was 0.011–0.051). This result revealed that supportive resources and achievement motivation partially mediate the effect of positive leadership on QWL.

**Table 6:** Bootstrap confidence interval estimation results of the simple mediating effect.

Variable relationship	Effect type	Effect value	LLCI	ULCI	Supported hypothesis
Positive leadership→ Supportive resources→QWL	Total effect	0.545	0.458	0.635	H2, H3
	Direct effect	0.277	0.200	0.353	
	Indirect effect	0.268	0.192	0.350	
Positive leadership→ Achievement motivation→QWL	Total effect	0.380	0.287	0.475	H4, H5
	Direct effect	0.277	0.200	0.353	
	Indirect effect	0.103	0.043	0.173	
Positive leadership →Supportive resources →Achievement motivation→ QWL	Total effect	0.467	0.375	0.562	H4,H6,H3
	Direct effect	0.277	0.200	0.353	
	Indirect effect	0.190	0.137	0.252	

LLCI represents lower limit 95% confidence intervals; ULCI represents upper limit: 95% confidence intervals.

### 4.4. Analysis of moderating effect

We adopted hierarchical linear regression analysis with three steps in this section to, respectively, test the moderating effect of culture on the relationship between positive leadership and achievement motivation (route a), the relationship between positive leadership and QWL (route b), and the relationship between positive leadership and supportive resources (route c). Step 1 employed control variables in the univariate analysis, and step 2 added independent variables (positive leadership) and moderating variables (culture) based on the former. Then, with the foundation of step 1 and step 2, step 3 drew into the interaction of independent variables and moderating variables (positive leadership^*^culture). Additionally, multicollinearity between the variables was asserted by giving all variables centered before testing. In the three steps of each moderation route, the maximum variance expansion factor is 4.619, which is lower than 10, suggesting that no multicollinearity existed. Thus, the results of the research are credible.

In step 1, the influence of the control variables on the dependent variable can be observed. Educational level and income had positive effects on QWL among the three routes. In terms of step 2, the influence of the independent variable (positive leadership: *β*_(a)_ = 0.238, *p*_(a)_ < 0.001; *β*_(b)_ = 0.349, *p*_(b)_ < 0.001; *β*_(c)_ = 0.454, *p*_(c)_ < 0.001) and moderating variable (culture: *β*_(a)_ = 0.443, *p*_(a)_ < 0.001; *β*_(b)_ = 0.500, *p*_(b)_ < 0.001; *β*_(c)_ = 0.333, *p*_(c)_ < 0.001) on the three dependent variables were significant. For step 3, the interaction of positive leadership and culture had a positive effect on achievement motivation (*β*_(a)_ = 0.192, < 0.001), QWL (*β*_(b)_ = 0.215, *p*_(b)_ < 0.001) and supportive resources (*β*_(c)_ = 0.195, *p*_(c)_ < 0.001), proving that culture has a moderating effect on all three paths (see Tables 7–9 for details).

**Table 7:** Moderating effect of culture on the relationship between positive leadership and achievement motivation.

Variable	Step 1			Step 2			Step 3		
	Dependent variable for steps 1–3: Achievement motivation								
	*β*	SE	*t*	*β*	SE	*t*	*β*	SE	*t*
Control variables									
Gender	−0.089	0.124	−2.271^*^	−0.063	0.102	−1.948	−0.065	0.099	−2.088^*^
Age	−0.091	0.107	−1.355	−0.024	0.088	−0.435	−0.008	0.085	−0.155
Marital status	−0.013	0.103	−0.3	−0.024	0.085	−0.67	−0.024	0.082	−0.684
Education level	−0.063	0.081	−1.566	0.001	0.067	0.042	−0.001	0.065	−0.027
Post	0.109	0.036	2.632^**^	0.052	0.029	1.519	0.056	0.029	1.691
Income	0.542	0.035	12.098^***^	0.114	0.036	2.452^*^	0.143	0.035	3.144^**^
Work shift	−0.108	0.104	−2.886^**^	−0.075	0.085	−2.413^*^	−0.068	0.083	−2.256^*^
Work seniority	−0.09	0.066	−1.36	−0.03	0.054	−0.555	−0.039	0.053	−0.752
Independent variables									
Positive leadership				0.238	0.06	3.815^***^	0.245	0.058	4.053^***^
Culture				0.443	0.064	7.089^***^	0.518	0.064	8.359^***^
Positive leadership × Culture							0.192	0.021	5.613^***^
*R* ^2^	0.392			0.591			0.617		
△*R*^2^	0.392			0.2			0.026		
*F*	37.338^***^			66.832^***^			67.633^***^		
VIF_max_	3.462			4.404			4.619		

^*^p < 0.05, ^**^p < 0.01, ^***^p < 0.001; VIF_max_, maximum variance expansion factor.

**Table 8:** Moderating effect of culture on the relationship between positive leadership and QWL.

Variable	Step 1			Step 2			Step 3		
	Dependent variable for steps 1–3: QWL								
	*β*	SE	*t*	*β*	SE	*t*	*β*	SE	*t*
Control variables									
Gender	−0.074	0.107	−2.058^*^	−0.042	0.067	−1.879	−0.045	0.062	−2.182^*^
Age	−0.116	0.092	−1.881	−0.033	0.058	−0.849	−0.015	0.053	−0.429
Marital status	0.018	0.089	0.452	0.003	0.056	0.102	0.003	0.051	0.125
Education level	−0.085	0.069	−2.341^*^	−0.005	0.044	−0.203	−0.007	0.04	−0.34
Post	0.085	0.031	2.243^*^	0.013	0.019	0.547	0.018	0.018	0.805
Income	0.613	0.03	14.98^***^	0.081	0.024	2.476^*^	0.112	0.022	3.746^***^
Work shift	−0.086	0.089	−2.511^*^	−0.045	0.056	−2.09^*^	−0.037	0.052	−1.885
Work seniority	−0.068	0.057	−1.13	0.006	0.036	0.155	−0.005	0.033	−0.133
Independent variables									
Positive leadership				0.349	0.04	8.012^***^	0.357	0.036	8.941^***^
Culture				0.5	0.043	11.446^***^	0.584	0.04	14.250^***^
Positive leadership × Culture							0.215	0.013	9.480^***^
*R* ^2^	0.492			0.8			0.833		
△*R*^2^	0.492			0.308			0.033		
*F*	56.113^***^			184.780^***^			208.469^***^		
VIF_max_	3.462			4.404			4.619		

^*^p < 0.05, ^**^p < 0.01, ^***^p < 0.001; VIF_max_, maximum variance expansion factor.

**Table 9:** Moderating effect of culture on the relationship between positive leadership and supportive resources.

Variable	Step 1			Step 2			Step 3		
	Dependent variable for steps 1–3: Supportive resources								
	*β*	SE	*t*	*β*	SE	*t*	*β*	SE	*t*
Control variables									
Gender	−0.052	0.142	−1.272	−0.027	0.111	−0.83	−0.029	0.107	−0.937
Age	−0.137	0.122	−1.939	−0.062	0.096	−1.128	−0.046	0.093	−0.864
Marital status	0.038	0.118	0.81	0.019	0.092	0.517	0.019	0.089	0.543
Education level	−0.132	0.092	−3.143^**^	−0.054	0.073	−1.651	−0.057	0.07	−1.778
Post	0.08	0.041	1.841	0.012	0.032	0.34	0.016	0.031	0.477
Income	0.455	0.04	9.699^***^	−0.035	0.039	−0.76	−0.006	0.038	−0.14
Work shift	−0.077	0.119	−1.963	−0.042	0.093	−1.369	−0.035	0.09	−1.176
Work seniority	−0.049	0.076	−0.706	0.019	0.059	0.351	0.009	0.057	0.18
Independent variables									
Positive leadership				0.454	0.065	7.325^***^	0.461	0.063	7.689^***^
Culture				0.333	0.07	5.362^***^	0.409	0.069	6.651^***^
Positive leadership × Culture							0.195	0.023	5.735^***^
*R* ^2^	0.332			0.597			0.623		
△*R*^2^	0.332			0.264			0.027		
*F*	28.887^***^			68.303^***^			69.369^***^		
VIFmax	3.462			4.404			4.619		

^*^p < 0.05, ^**^p < 0.01, ^***^p < 0.001; VIF_max_, maximum variance expansion factor.

**Figure 3:**
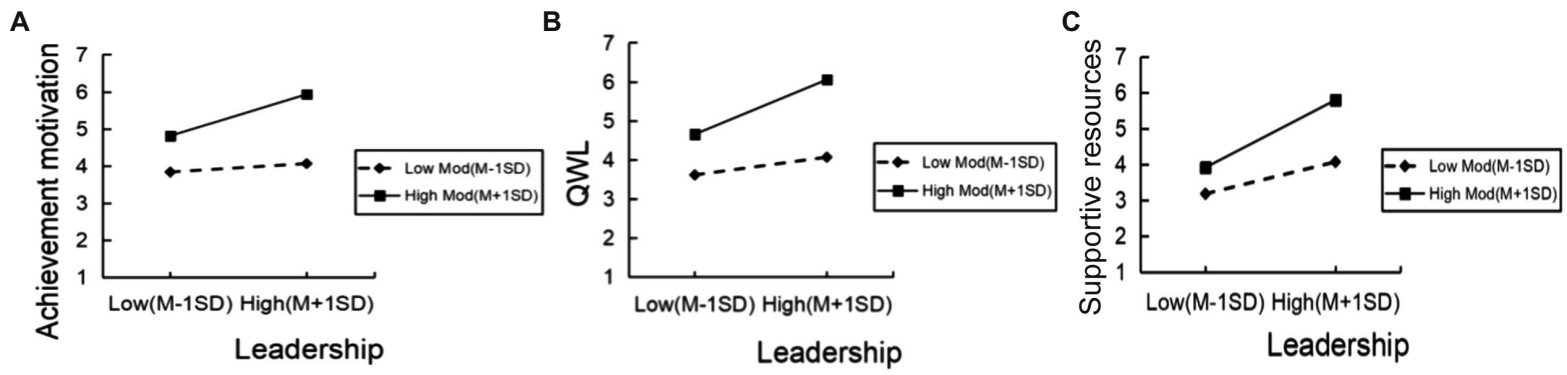
Moderating effect diagram of culture.

In addition, we used a simple slope test to further explore the moderating roles of culture in the three paths. First, it is verified that culture has a considerable impact on the relationship between positive leadership and achievement motivation, the relationship between leadership and QWL, and the relationship between positive leadership and supportive resources. Second, the interaction diagram (Figure 3) apparently reports the moderation effects of culture on the three routes, which indicates the interaction of positive leadership and culture at high (mean + 1SD) and low (mean – 1SD) levels of the culture. The slope values delegate the impact of positive leadership on achievement motivation (shown in Figure 3A), QWL (shown in Figure 3B) and supportive resources (shown in Figure 3C). The results indicated that the moderating effects were more significant and active if the respondents perceived a higher culture in the organization.

In summary, the moderating effect of culture was significant on the route of positive leadership-supportive resources (route c) in the high-culture perception group (*β*_(c)_ = 0.721, *t*_(c)_ = 10.230, *p*_(c)_ < 0.001) and low-culture perception group (*β*_(c)_ = 0.201, *t*_(c)_ = 2.901, *p*_(c)_ = 0.004). However, in route a (positive leadership – achievement motivation) and route b (positive leadership – QWL), the moderating effect was positive in the high-culture perception group (*β*_(a)_ = 0.501, *t*_(a)_ = 7.717, *p*_(a)_ < 0.001; *β*_(b)_ = 0.643, *t*_(b)_ = 15.887, *p*_(b)_ < 0.001), but had no positive effect in the low-culture perception group (*β*_(a)_ = −0.011, *t*_(a)_ = −0.169, *p*_(a)_ = 0.866; *β*_(b)_ = 0.071, *t*_(b)_ = 1.771, *p*_(b)_ = 0.077).

### 4.5. Analysis of moderated mediation effect

We applied Model 85 in the PROCESS macro with 5,000 bootstrap samples to test moderated mediation analysis. The moderated mediation effect of positive leadership on QWL *via* achievement motivation and supportive resources at distinct scores of culture was examined. As shown in Table 10, the moderated mediation effects were positive in all of the high level (mean + 1 SD), mean and low level (mean – 1 SD) respondents, as confirmed by the bootstrap 95% CI. Furthermore, the index was statistically significant (*β* = 0.013), and the bootstrap 95% CI (0.007–0.020) excluded 0, revealing that culture had a positive moderated mediation effect on the relationship between positive leadership and QWL through achievement motivation and supportive resources.

**Table 10:** Bootstrap confidence interval estimation results of moderated mediating effect: specific conditional values of culture and the index of moderated mediation.

Culture	*β*	SE	LLCI	ULCI
−1SD	0.030	0.009	0.012	0.049
Mean	0.047	0.010	0.028	0.068
+1SD	0.063	0.013	0.040	0.090
	Index	SE	LLCI	ULCI
Culture	0.013	0.004	0.007	0.020

LLCI represents lower limit 95% confidence intervals; ULCI represents upper limit: 95% confidence intervals.

## 5. Discussion

### 5.1. Practice implications and future prospects

This study proves the influence of positive leadership in promoting the QWL of family doctors, in the context of PCIC practice. The interactions of environmental factors, psychological factors and culture in the PCIC model are explored. SEM, hierarchical linear regression analysis, and simple slope test are adopted to analyze the data from 473 family doctors in representative counties of Zhejiang Province. The results of SEM indicate that positive leadership has a direct effect on QWL and a mediating effect on QWL through supportive resources and achievement motivation. The hierarchical linear regression analysis shows that culture has a positive moderating effect on the relationship between three pairs of variables: positive leadership and achievement motivation, positive leadership and QWL, and positive leadership and supportive resources. The simple slope test proves that culture has a moderated mediating effect on the relationship between positive leadership and QWL *via* supportive resources and achievement motivation.

When analyzing demographic characteristics, we found significant differences in the QWL of different groups of family doctors. First, the younger group has a better experience of QWL, which is consistent with existing research, such as Klaghofer et al. (2010) and Penberthy et al. (2018), showing that younger doctors are more likely to maintain higher levels of professional enthusiasm and self-confidence, with less emotional burnout. In other words, among older family doctors, perceiving a high level of QWL is more difficult, suggesting that the psychological traits of older staff need to be considered when promoting QWL. In addition, married family doctors had the highest QWL score, which is in line with the extant literature: for instance, Tawfik et al. (2021) proved that married doctors had better work-life integration. Therefore, more attention should be given to family doctors to improve their work-life balance. However, there is an interesting finding that family doctors with lower education levels had better QWL, which is contrary to the findings of many studies on the correlation between high education levels and good QWL (Golubic et al., 2009). This phenomenon may be caused by the special professional environment of family doctors in China. Influenced by marketization since the 1980s, the CHCs in China has gradually been at a disadvantage in competition with hospitals; therefore, adverse selection of medical personnel in CHCs has gradually formed (Hsieh and Tang, 2019). Medical staff with lower educational levels and lower job expectations may be recruited in CHCs, while those with higher educational level are more eager to work in hospitals rather than in CHCs. Therefore, improving the professional identity of highly educated family doctors is an entry point for improvement. Since demographic sociological characteristics form the basis of the intrinsic psychological characteristics of individuals, it suggests to some extent that psychological characteristics need to be taken into account in QWL improvement.

In this study, the SEM proved that positive leadership has positive effects on the QWL of family doctors, through the mediating effect of supportive resources. Positive leadership may provide family doctors with better rewards such as higher compensation, more career promotion opportunities and personal development opportunities; however, among these rewards, the resources supporting family doctor service play an indispensable role. These findings are supported by some existing research. For example, Luo et al. (2022) demonstrated that sufficient resources are needed in staff motivation for active engagement. In addition, the working environment may also be improved by positive leadership through supportive resources. For instance, sufficient drug supply and doctor–patient relationship-friendly policies and rules will provide family doctors with a more relaxed doctor–patient cooperative environment. de Oliveira Vasconcelos Filho et al. (2016) argued that available resources will be able to motivate health care teams to achieve better healthcare outcomes. All the aforementioned findings highlight the necessity of sectorial collaboration in resource delivery, such as the financial department, medical insurance system, education and training system, etc. Positive leadership is key in such collaboration, which plays the role of reasonable allocation, coordination and utilization of resources for QWL promotion.

The SEM also proved that individual psychological factors mediate the effect of positive leadership on the QWL of family doctors. Positive leadership may stimulate the achievement motivation of family doctors to pursue valuable goals, achieve high performance, and strive for success. A possible explanation is that, first, positive leadership may promote the satisfaction of working expectations by highlighting the strengths of each participant and emphasizing the value of the work. This is consistent with previous research results, that is, the positive leadership of the family doctor team will be able to create a value-driven organizational environment, promote integration and coordination among team members, and ultimately achieve high-quality family doctor services (Jenkins et al., 2021; Mathews et al., 2022). In addition, positive leadership may also foster working competence and increase self-efficacy by encouraging interprofessional collaboration among team members, such as multiple disciplinary team service. In addition, the working environment may also be improved, since positive leadership may stimulate the internal motivation of family doctors to pursue friendly interpersonal interactions. This result corresponds to a study on maternal care; that is, positive leadership has created a patient-centered collaborative nursing model in family doctor care, which emphasizes harmonious interpersonal interaction to achieve efficient family doctor care (Pecci et al., 2007). Furthermore, there are complex interactions of supportive resources-achievement distant mediating effects. Available resources such as funding may stimulate the achievement motivation of family doctors under a reasonably value-oriented compensation system guided by positive leadership, thus further improving the QWL of family doctors.

In addition, the moderating model provided a more detailed explanation of the influence by which positive leadership promotes QWL among family doctors. We found significant positive mediating effects of supportive resources and achievement motivation between positive leadership and QWL, which can be moderated by culture. This suggests that culture is conducive to helping leaders to better coordinate resources and motivate staff engagement. The better the culture-shaping in the family doctor team, the more effective promotion of QWL will be gained. This is consistent with Kim et al.’s finding that a supportive culture changes employees’ attitudes and self-efficacy and ultimately has a positive impact on work and quality of life (Kim and Jang, 2018). It is also worth mentioning that on the pathway mediated by achievement motivation, there was a significant positive moderating effect only for high culture subgroup, but not low culture subgroup. This result demonstrated that a certain high level of people-centered culture is required to moderate QWL.

### 5.2. Strengths and limitations

This study proves the effects of positive leadership on family doctor QWL, with the interactions of environmental factors, organizational factors and individual psychological factors. It provides a theoretical basis for the co-governance of multiple participants in family doctor services with stakeholders of the government, healthcare organizations, family doctor teams and individuals. The study also explores the role of culture in the context of PCIC, which may provide evidence for the potential influences of PCIC practice on the positive leadership and QWL of medical staff.

A series of measurements have been taken to improve the research quality. First, an extensive search of multiple databases was conducted in the literature study to ensure that existing developed measurement scales in the published literature were adequately included. Second, informed interviews and Delphi consultations were also used to revise the measurement scale to ensure that the QWL of family doctors could be accurately evaluated by measurement in the context of the local sociocultural environment. Third, multiple statistical analysis methods were adopted to mutually confirm the effect of positive leadership on family doctor QWL; for example, the moderating effect of culture was verified by hierarchical regression and a simple slope test.

The possible limitations in this study are as follows. First, response bias cannot be excluded due to data collection with self-report measures, and the measurements were based on personal experience rather than objective data, although we installed control variables to avoid individual interference. Second, there is a bias in the average annual income of individuals. This is because the reference base we used is based on the overall level of China, while most medical staff in Zhejiang Province belong to the high-income group. Third, the questionnaire used in this study was self-designed, and the quality of measurement needs special evaluation. However, the indicators were derived from a series of questionnaires validated by several studies and with reference to interview results and the Delphi method, so the questionnaire has good reliability and validity. Fourth, this study is cross-sectional and does not measure the impact of change over time. However, family doctor service policies were relatively stable during this period. Last, the results may not be representative of other regions because all participants were sampled in Zhejiang Province in China. However, since Zhejiang Province is the pilot area of PCIC, family doctor teamwork, positive leadership and culture in such an environment will still provide a reference for other countries facing similar challenges of primary health care failure and low enthusiasm of family doctors in similar settings.

It is worth further discussing that extended research aspects could focus on the influencing factors of positive leadership rather than just the role of positive leadership. Clearly, the main reason for poor QWL is inadequate positive leadership, but which factors influence positive leadership can be explored in subsequent studies. Additionally, in this study, we can also combine the positive leadership of primary healthcare with the relevant theories of different leadership styles to further explore whether different leadership styles promote inspirational motivation in the family doctor team and explore the changes in the QWL of family doctors under different leadership styles. This means it could ensure that the impact of act motivation related to family doctors on perceived QWL can be tested. Ultimately, this study would be able to expand the sample size in domestic regions and then conduct multiple group analyses and comparisons to determine if the influence of QWL differs across regions.

## 6. Conclusion

This study proved the influence of positive leadership on the QWL of family doctors. QWL can be significantly improved by positive leadership in the context of PCIC practice, among which supportive resources and achievement motivation mediate the positive correlation between positive leadership and QWL. In addition, people-centered culture has a moderate mediating influence on the relationship between positive leadership and QWL through supportive resources and achievement motivation. Moreover, positive leadership had a significant effect on QWL for the high-culture perception group, but had no effect for the low-culture perception group. In summary, the interaction among multiple factors, including environmental factors, individual physiological features and culture, may influence the positive effects of positive leadership on the QWL of family doctors in the context of PCIC.

## Data availability statement

The original contributions presented in the study are included in the article/Supplementary material, further inquiries can be directed to the corresponding author.

## Ethics statement

The studies involving human participants were reviewed and approved by Hangzhou Normal University Scientific Research Ethics Committee (approval number: 20190022). The patients/participants provided their written informed consent to participate in this study.

## Author contributions

WS: formal analysis, writing—original draft preparation, and validation. XH: conceptualization, methodology, and software. XY and YW: writing—original draft preparation and validation. ZH: investigation and data collection. YC, CP, and YJ: review and data collection. All authors contributed to the article and approved the submitted version.

## Funding

This work was supported by: (1) the National Natural Science Foundation of China (grant number: 72004051)–[Simulation study on the optimization strategy of medical and prevention integration and community linkage governance of medical association in response to major epidemics]; (2) Humanities and Social Sciences Fund of Ministry of Education of China (Project No.: 18YJCZH232)–[Research on the Motivation and Promotion Strategy of Family Doctors’ signing Service].

## Conflict of interest

The authors declare that the research was conducted in the absence of any commercial or financial relationships that could be construed as a potential conflict of interest.

## Publisher’s note

All claims expressed in this article are solely those of the authors and do not necessarily represent those of their affiliated organizations, or those of the publisher, the editors and the reviewers. Any product that may be evaluated in this article, or claim that may be made by its manufacturer, is not guaranteed or endorsed by the publisher.
